# A multicenter case–control study of the effect of e-nos VNTR polymorphism on upper gastrointestinal hemorrhage in NSAID users

**DOI:** 10.1038/s41598-021-99402-w

**Published:** 2021-10-07

**Authors:** Narmeen Mallah, Maruxa Zapata-Cachafeiro, Carmelo Aguirre, Eguzkiñe Ibarra-García, Itziar Palacios–Zabalza, Fernando Macías García, Julio iglesias García, María Piñeiro-Lamas, Luisa Ibáñez, Xavier Vidal, Lourdes Vendrell, Luis Martin-Arias, María Sáinz Gil, Verónica Velasco-González, Ángel Salgado-Barreira, Adolfo Figueiras

**Affiliations:** 1 Department of Preventive Medicine, University of Santiago de Compostela, Santiago de Compostela, Spain; 2 Consortium for Biomedical Research in Epidemiology and Public Health (CIBER en Epidemiología Y Salud Pública- CIBERESP), Carlos III Health Institute, Madrid, Spain; 3 Health Research Institute of Santiago de Compostela (IDIS), Santiago de Compostela, Spain; 4 Biocruces Bizkaia Health Research Institute, Pharmacotherapy Group, Bizkaia, Spain; 5 University Hospital of Galdakao-Usansolo. Basque Country Pharmacovigilance Unit, Osakidetza, Spain; 6 Pharmacology Department, Medicine and Nursing Faculty, University of the Basque Country, Barakaldo, Bizkaia Spain; 7 Pharmacy Department, Osakidetza Basque Health Service, Urduliz Hospital, Urduliz, Spain; 8 Department of Gastroenterology and Hepatology, University Hospital of Santiago de Compostela, Santiago de Compostela, Spain; 9 Department of Pharmacology, Therapeutics and Toxicology, Clinical Pharmacology Service, Catalonian Institute of Pharmacology, Vall d’Hebron University Teaching Hospital, Autonomous University, Barcelona, Spain; 10 Centre for Research On Drug Safety (CESME), Valladolid University, Valladolid, Spain

**Keywords:** Gastroenterology, Pharmacogenetics

## Abstract

Bleeding in non-steroidal anti-inflammatory drug (NSAID) users limited their prescription. This first multicenter full case–control study (325 cases and 744 controls), explored the association of e-NOS intron 4 variable number tandem repeat (VNTR) polymorphism with upper gastrointestinal hemorrhage (UGIH) in NSAID exposed and unexposed populations and assessed any interaction between this polymorphism and NSAIDs. NSAID users carrying e-NOS intron 4 wild type genotype or VNTR polymorphism have higher odds of UGIH than those unexposed to NSAIDs [Odds Ratio (OR): 6.62 (95% Confidence Interval (CI): 4.24, 10.36) and OR: 5.41 (95% CI 2.62, 11.51), respectively], with no effect modification from VNTR polymorphism-NSAIDs interaction [Relative Excess Risk due to Interaction (RERI): −1.35 (95% CI −5.73, 3.03); Synergism Index (S): 0.77 (95% CI 0.31, 1.94)]. Similar findings were obtained for aspirin exposure. Non-aspirin NSAID users who carry e-NOS intron 4 VNTR polymorphism have lower odds of UGIH [OR: 4.02 (95% CI 1.85, 8.75) than those users with wild type genotype [OR: 6.52 (95% CI 4.09, 10.38)]; though the interaction estimates are not statistically significant [RERI: −2.68 (95% CI −6.67, 1.31); S: 0.53 (95% CI 0.18, 1.55)]. This exploratory study suggests that the odds of UGIH in NSAID or aspirin users does not modify according to patient´s e-NOS intron 4 genotype.

## Introduction

Aspirin and non-aspirin non-steroidal anti-inflammatory drugs (NSAIDs) are among the most frequently used therapeutic agents for a wide variety of indications due to their effectiveness and cost-efficiency^1–3^. The use of aspirin, an archetype of NSAIDs, in secondary prevention against cardiovascular diseases is well established^4^. Recent reports also demonstrated that the beneficial effects of NSAIDs and aspirin extend to a reduced risk of other major diseases including digestive tract cancers, hepatocellular carcinoma, cholangiocarcinoma, breast cancer, pancreatitis and liver fibrosis^5–10^. However, the idiosyncratic effects manifested by susceptible individuals, mainly gastrointestinal bleeding, have limited the application of NSAIDs and aspirin in primary prevention^11–13^. Gastrointestinal bleeding remains a major medical concern as it is among the main causes of hospitalizations related to digestive diseases in several countries^14^. Non-variceal upper gastrointestinal hemorrhage (UGIH) represents a serious medical challenge worldwide, with an incidence of 48 to 160 cases per 100,000 adults per year and a mortality rate ranging between 1.1% and 11%^15^.

Nonetheless, a general discontinuation of NSAIDs or aspirin use might not be a sufficient solution. A recent cohort study of low-dose aspirin for primary or secondary prevention against cardiovascular diseases showed that the cessation of aspirin therapy in patients free from major surgery or bleeding increases the risk of cardiovascular events by more than 30%^16^. Accordingly, a combination of pharmacogenetic and pharmacoepidemiologic studies is needed to assess and improve the use of personalized NSAIDs treatment.

Gastrointestinal bleeding in patients who use NSAIDs and aspirin was associated with genetic polymorphisms^17–22^. In the gastrointestinal tract, vasodilation and mucosal blood flow are mediated by Nitric Oxide (NO). Prostaglandins also exert a fundamental role in protecting the gastric mucosa from damage^23^. Circulating NO is mainly produced by endothelial NO synthase (e-NOS) and plays a fundamental role in protecting the gastric mucosa, repairing gastric damage induced by NSAIDs and aspirin, regulating blood flow, fostering angiogenesis, preventing thrombosis, cardiac or vascular contractility, controlling cardiovascular tissue remodeling, and inhibiting platelet aggregation^24–27^. Reduced bioavailability of NO predisposes to hypertension, thrombosis and vasospasm^28^.

Genetic variations in e-NOS influence the expression of this gene, and thus could modify drug response and influence the development of some diseases^24,26,29^. In specific, the levels of NO in the plasma are significantly affected by genetic variations in intron 4 of e-NOS gene^30^. The 27-base pairs (bp) variable number of tandem repeat (VNTR) polymorphism in this intron represents a type of these genetic variations. VNTRs are sequences of DNA with a high degree of polymorphism among individuals in the length of repeated nucleotides that lie adjacent to each other in the genome^31^. The ancestral allele of e-NOS intron 4 VNTR polymorphism consists of five repeats of the 27 bp segment and is known as “4b”, whereas its variant comprises a tandem of four repeats of the 27 bp sequence and is denoted “4a”^24,30^.

The allele 4a was previously associated with a lower risk of UGIH in aspirin users^32^. Nonetheless, the protective effect of this e-NOS genetic variant against UGIH was determined in a study that included a selective group of patients exposed to aspirin^32^. The lack of data about individuals unexposed to aspirin in that study^32^, makes it impossible to determine whether the reported reduced risk of UGIH is due to a protective effect of 4a e-NOS allele itself or generated from a modification of effect from an interaction between this genetic variation and aspirin, in which case, further investigation is needed.

Accordingly, in this study, we aimed to explore the association of e-NOS intron 4 VNTR polymorphism with UGIH in a population composed of patients exposed and unexposed to NSAIDs. In addition, we investigated the effect modification by 4a and 4b alleles on NSAIDs-related UGIH. We also performed a stratified analysis by type of NSAIDs (i.e. any NSAIDs, aspirin NSAIDs and non-aspirin NSAIDs).

2. Methods.

### Study design and population

We carried out a multicenter, full case–control study. Three hundred twenty-five cases and 744 controls were prospectively recruited (incident cases) from four health centers in Spain in two different waves (2004–2007, and 2013–2015).

Cases were defined as patients admitted to the hospital for UGIH, diagnosed surgically or endoscopically. All eligible UGIH cases of any grade of severity were included.

Controls were patients who had a programmed unpainful surgery (such as prostatic adenoma, eye cataract, ear pinning, lipoma, vocal cord cyst, septoplasty and thyroid nodules) that was not related to NSAID exposure.

Adults (> 18 years old), of European origin, and biologically unrelated cases and controls were recruited from the same health centers. We used the native tongue of the participants or the original language of their parents as a proxy of ethnicity^33–36^.

Cases and controls were matched on age, sex, recruiting health center and period of recruitment. We used the same protocol as that of two related studies published previously^21,37^.

Participants were interviewed by trained health personnel using a comprehensive questionnaire that was specifically designed for this study. The questionnaire included questions about the consumed medicines, source of prescriptions and indications of use, recurrent symptoms for which NSAIDs were recommended and the prescribed treatments to alleviate these symptoms. It also included questions about caffeine consumption, dietary habits, alcohol intake and tobacco smoking. Prompt cards of commercial NSAIDs (aspirin and non-aspirin NSAIDs) boxes were used to help the participant identify and recall better the use of these drugs. All NSAIDs were considered for the analysis, irrespective of their route of administration. The interview was repeated on subsequent dates when needed, such as in case of the patient’s tiredness to complete the whole interview and/or failure to remember some information.

The reliability of the interview was rated using a 0–10 Likert scale as perceived by the interviewer. To confirm that NSAID exposure took place prior to any symptom of UGIH, an index date of NSAID use was defined. For cases, the index date was the day of occurrence of the first symptoms of UGIH, whereas it was the recruitment date for the controls. We determined the index date by relying on the patient’s clinical history, but the researchers were blind to patient’s exposure to NSAIDs. In line with previous related studies, a seven-day etiologic window was established starting from the index date^38–40^.

All participants were tested for the presence of anti-*Helicobacter pylori* immunoglobulin G using enzyme linked immunosorbent assay (ELISA). The following commercial kits were applied according to the manufacturer’s protocol [Human Anti-*Helicobacter pylori* IgG ELISA Kit (ab108736, Abcam, Cambridge, England), and Captia™ *H. pylori* IgG EIA (ref: 2,346,400, Trinity Biotech Captia, Co. Wicklaw, Ireland)]. Participants were asked whether they have ever been treated against *Helicobacter pylori* infection in order to avoid having any false positive result from a previous infection.

### Genotyping

The eNOS intron 4 VNTR polymorphism was detected by polymerase chain reaction (PCR) using the following primers: 5′-AGGCCCTATGGTAGTGCCTTT-3′ and 5′-TCTCTTAGTGCTGTGGTCAC-3′^41–43^. The reaction mixture (a total volume of 25 µl) contained 2.5 µl of 10 × PCR buffer, including 100 ng of genomic DNA, 1.5 mM MgCl2, 0.2 mM of each dNTP, 500 nmol of each primer and 5 U Taq polymerase. PCR was performed with 35 cycles of 30 s at 94 °C for denaturation, 30 s at 63 °C for annealing, and 1 min at 72 °C for extension, followed by final extension for 5 min at 72 °C. The following fragments were produced: 393 bp band (4 copies of the 27 bp repeat), and 420 bp band (5 copies of the 27 bp repeat). The amplified product was size fractionated by the D1000 Screen Tape assay (Agilent Technologies, CA) on Agilent 4200 TapeStation system according to manufacturer’s recommendations. For quality control, 5% of the samples were selected to repeat PCR and genotyping, and no discrepancies were found. All cases and controls were genotyped using a phenotype-blind process. Hardy–Weinberg equilibrium was tested in the control group (significance threshold: P-value < 0.001), using SNPassoc Library of the R package^44^, to check for possible bias in the selection of controls^45,46^.

### Statistical analysis

The 27 bp e-NOS intron 4 VNTR polymorphism was genotyped for all participants who were then stratified into four categories according to NSAID exposure (exposed/unexposed) and genotype (wild type/genetic variation). The wild type genotype was denoted “b allele” and defined as five copies of the 27 bp repeats in both alleles (5/5). The genetic variant was denoted “a allele” when characterized by four copies of the 27 bp repeats in both alleles (4/4) or “ab allele” in case of possessing four copies of the 27 bp repeats in one allele and five copies of the same repeat in the second allele (4/5). The group that comprised patients who were “unexposed to NSAIDs and carriers of the wild type genotype” was used as a reference category.

Adjusted Odds Ratios (OR) of UGIH and their 95% Confidence Intervals (CI) were estimated using the generalized linear mixed models for dependent binomial variables. The statistical models were constructed by considering the following four consecutive levels: patient, strata of cases and controls (each case and its matched controls), health center, and period of patients’ recruitment. We used a random-effects model to assess the effect of the period of patients’ recruitment and a nested random-effects model for the strata of cases and controls, and health center. The models were estimated using the lmer function of the lme4 R package^47^. Initially, in univariate analysis, we explored the effect of potential confounders including age, Body Mass Index (BMI), sex, tobacco smoking, alcohol intake, caffeine consumption, diet, previous history of arthrosis and/or gastrointestinal disorders other than UGIH, *Helicobacter pylori* infection, source of information (i.e. whether the interview was answered by the patient independently or with the help of a healthcare assistant / direct relatives), number of undertaken interviews, reliability of the interview as perceived by the interviewer, and exposure to medications that are not NSAIDs. Potential confounders were retained in the model if they changed the OR of the main variable by at least 10%, and provided that the Schwartz’s Bayesian Information Criterion was enhanced^48,49^.

Subsequently, the additive interaction effect between e-NOS intron 4 VNTR polymorphism and NSAIDs on UGIH was determined by calculating the Relative Excess Risk due to Interaction (RERI) and the Synergism Index (S) estimates along with their 95% CI.

We also undertook a stratified analysis by type of NSAIDs where we assessed the effect of e-NOS VNTR polymorphism on UGIH in 1) non-aspirin NSAIDs users, and 2) aspirin NSAIDs users.

### Ethical aspects

Participants signed a written informed consent before their enrolment in the study.

### Ethics approval

The study was approved by the ethics committee for clinical research of each participating health center: Barcelona (CEIC; protocol number: Es38121226Z), Euskadi (CEIC-E; protocol number: PI2013101), Galicia (CEIC-G; protocol number: 2013/263) and Valladolid (CEIC-VA-ESTE-HCUV; protocol number: PI-14–142). All procedures were performed in accordance with the 1964 Helsinki declaration and comparable ethical standards.

## Results

### General patients’ characteristics

Three hundred twenty-five cases and 744 controls fulfilled the inclusion criteria and were included in the study. Their demographic and clinical characteristics are presented in Table 1.

**Table 1:** Clinical and demographic characteristics of the study population**.** CI, confidence interval; N, number of participants; OR, odds ratio.

Characteristic	Cases (N = 325) N (%)	Controls (N = 744) N (%)	OR (95% CI)
**Study period**			
EMPHOGEN 1 (2004–2007)	253 (77.8%)	590 (79.3%)	0.71 (0.51, 0.99)
EMPHOGEN 2 (2013–2015)	72 (22.2%)	154 (20.7%)	1
**Age**			
< 45	41 (12.6%)	95 (12.8%)	1
45–65	116 (35.7%)	267 (35.9%)	1.06 (0.68, 1.65)
> 65	161 (49.5%)	370 (49.7%)	1.01 (0.66, 1.54)
Missing	7 (2.2%)	12 (1.6%)	–
**BMI**			
Underweight	10 (3.1%)	24 (3.2%)	0.73 (0.33, 1.61)
Normal weight	113 (34.8%)	202 (27.2%)	1
Overweight	128 (39.4%)	372 (50.0%)	0.61 (0.44, 0.83)
Obesity	68 (20.9%)	144 (19.4%)	0.84 (0.57, 1.23)
Missing	6 (1.8%)	2 (0.3%)	–
**Sex**			
Male	235 (72.3%)	556 (74.7%)	1
Female	87 (26.8%)	188 (25.3%)	1.18 (0.87, 1.61)
Missing	3 (0.9%)	0	–
**Arthrosis**			
No	218 (67.1%)	466 (62.6%)	1
Yes	86 (26.5%)	215 (28.9%)	0.86 (0.63, 1.17)
Missing	21 (6.5%)	63 (8.5%)	
***Helicobacter pylori***			
No or inconclusive	27 (8.3%)	136 (18.3%)	1
Yes	275 (84.6%)	572 (76.9%)	2.52 (1.60, 3.96)
Missing	23 (7.1%)	36 (4.8%)	
**Source of information**			
Patients	258 (79.4%)	668 (89.8%)	1
Healthcare assistants / direct relatives	67 (20.6%)	76 (10.2%)	2.34 (1.61, 3.41)
**Interview variables** **Number of interviews conducted**			
1	273 (84.0%)	640 (86.0%)	1
≥ 2	52 (16.0%)	104 (13.9%)	1.27 (0.81, 2.00)
**Reliability of the interview**			
< 5	13 (4.0%)	20 (2.7%)	1
5–7	36 (11.1%)	77 (10.3%)	0.68 (0.30, 1.55)
7–9	134 (41.2%)	310 (41.7%)	0.70 (0.33, 1.48)
≥ 9	142 (43.7%)	337 (45.3%)	0.67 (0.32, 1.41)
**Personal history of gastrointestinal disorders**			
None or dyspepsia	207 (63.7%)	644 (86.6%)	1
Ulcer	48 (14.8%)	55 (7.4%)	2.78 (1.81, 4.27)
Bleeding	70 (21.5%)	45 (6.0%)	4.77 (3.14, 7.25)
**Exposure to other medications**			
Inhibitors of COX2	3 (0.9%)	6 (0.8%)	0.88 (0.21, 3.75)
Analgesics not narcotics	54 (16.6%)	56 (7.5%)	2.72 (1.80, 4.13)
Inhibitors of the proton pump	36 (11.1%)	65 (8.7%)	1.21 (0.77, 1.90)
Antiaggregant	65 (20.0%)	86 (11.6%)	1.93 (1.33, 2.79)
Anticoagulants	35 (10.8%)	32 (4.3%)	3.07 (1.84, 5.13)

### Genotyping

The genotyping was successful for all 1069 participants. Table 2 represents the distribution of the wild type and the polymorphic genotypes across cases and controls.

**Table 2:** Distribution of wild type and polymorphic alleles of e-NOS intron 4 VNTRs polymorphism across cases and controls.

Genotype	Cases (N = 325) N (%)	Controls (N = 744) N (%)
**Wild type**		
homozygous “b” (420 bp/420 bp)	247 (76%)	550 (73.9%)
**Genetic variation**		
heterozygous “ab” (393 bp/420 bp)	67 (20.6)	167 (22.4%)
homozygous “a” (393 bp/393 bp)	8 (2.5%)	23 (3.1%)
Others^a^	3 (0.9%)	4 (0.5%)

^a^ Others group included the following genetic variations: 420 bp/447 bp (3 cases and 2 controls), 366 bp/420 bp (1 control) and 370 bp/420 bp (1 control).

The homozygous genotype (420 bp/420 bp) denoted by “b” allele was the most prevalent in our study population [247 cases (76%) and 550 (73.9%) controls] (Table 2). Accordingly, “b” allele was deemed as the ancestral wild type allele.

Seventy-eight (24%) cases and 194 (26.1%) controls carried genetic variants of e-NOS intron 4 VNTR polymorphism. The controls followed Hardy–Weinberg equilibrium (p-value = 0.025).

### A*ssociation of e-NOS VNTR polymorphism with UGIH*

Table 3 summarizes the association of NSAID, non-aspirin NSAID and aspirin use with UGIH, stratified by e-Nos intron 4 genotype (wild type/genetic variation).

**Table 3:** Effect of e-NOS intron 4 VNTR polymorphism and NSAIDs exposure on the risk of UGIH.

NSAID Exposure	Wildtype genotype		Genetic variation		RERI (95% CI)	S (95% CI)
	Cases/controls N/N	OR (95% CI) ^a^; *p* value	Cases/controls N/N	OR (95% CI) ^a^ *p* value		
**All NSAIDs**						
No use	144/465	1.00	51/169	1.14 (0.74, 1.74) p = 0.5514	−1.35 (−5.73, 3.03)	0.77 (0.31, 1.94)
Use	103/85	6.62 (4.24, 10.36) p < 0.0001	27/25	5.41 (2.62, 11.15) p < 0.0001		
**Non-aspirin NASIDs**						
No use	159/478	1.00	56/171	1.18 (0.78, 1.78) p = 0.4297	−2.68 (−6.67, 1.31)	0.53 (0.18, 1.55)
Use	88/72	6.52 (4.09, 10.38) p < 0.0001	22/23	4.02 (1.85, 8.75) p = 0.0005		
**Aspirin NSAIDs**						
No use	221/537	1.00	72/190	1.03 (0.70–1.51) p = 0.8715	0.60 (−8.96, 10.17)	1.14 (0.15, 8.92)
Use	26/13	5.17 (2.11–12.68) p = 0.0003	6/4	5.80 (1.32–25.46) p = 0.0197		

CI, confidence interval; N, number of participants; OR odds ratio; RERI, Relative Excess Risk due to Interaction; S, Synergism Index. ^a^ Odds Ratio adjusted for: period of patients’ recruitment, previous history of arthrosis, infection with *Helicobacter pylori*, gastrointestinal disorders (ulcer and bleeding), exposure to inhibitors of the proton pump, exposure to antiaggregant, exposure to anticoagulants, the number of conducted interviews, and the reliability of the interview. Aspirin subgroup analysis was additionally adjusted for exposure to non-aspirin NSAIDs. RERI = 0 and S = 1 imply no interaction, RERI > 0 and S > 1 imply positive interaction and RERI < 0 and S < 1 imply negative interaction.

No association was observed between the polymorphic alleles of e-NOS intron 4 VNTR and UGIH in patients unexposed to any NSAID [OR: 1.14 (95% 0.74, 1.74)], non-aspirin NSAIDs [OR: 1.18 (95% 0.78, 1.78)] and aspirin [OR: 1.03 (95% CI 0.70, 1.51)] (Table 3).

### Modification of the effect of NSAIDs on UGIH by e-NOS VNTR polymorphism

As shown in Table 3, an association was observed between exposure to any NSAID and UGIH. Nonetheless, no substantial difference in the magnitude of this association was detected between NSAIDs users with e-NOS intron 4 wild type genotype [OR: 6.62 (95% CI 4.24, 10.36)] and those users with e-NOS intron 4 VNTR polymorphism [OR: 5.41 (95% CI 2.62, 11.15)].

Similar findings were obtained for aspirin exposure by individuals carrying the wild type genotype [OR: 5.17 (95% CI 2.11, 12.68)] and by individuals carrying the genetic variants of e-NOS intron 4 [OR: 5.80 (95% CI 1.32, 25.46)] (Table 3).

Regarding exposure to non-aspirin NSAIDs, patients carrying e-NOS intron 4 VNTR polymorphism have lower odds of UGIH than non-aspirin NSAID users with wild type genotype of e-NOS intron 4 [OR: 4.02 (95% CI 1.85–8.75) and OR: 6.52 (95% CI 4.09, 10.38), respectively] (Table 3).

Our exploratory analysis suggests that there is no interaction between e-NOS intron 4 VNTR polymorphism and any NSAID on UGIH. We also did not observe an interaction between e-NOS intron 4 VNTR polymorphism and aspirin exposure. However, our findings reveal decreased odds of UGIH from a potential interaction between e-NOS intron 4 VNTR polymorphism and non-aspirin NSAID use, though the interaction estimates were not statistically significant [RERI: −2.68 (95% CI −6.67, 1.31); S: 0.53 (95% CI 0.18, 1.55)].

## Discussion

Numerous studies associated the risk of gastrointestinal bleeding with genetic polymorphisms. However, there is a lack of knowledge about the influence of genetic variation on UGIH from NSAID and aspirin exposure. eNOS gene is known to exert fundamental activities in protecting gastric mucosa and repairing NSAIDs/aspirin-caused damage. Nevertheless, there is a shortage of information about the effect of variations in this gene on drug response^26^. In a recent study on the effect of polymorphisms on aspirin-related UGIH, we suggested that aspirin users who carry the inherited allele of rs1799983 polymorphism of NOS3 gene have lower odds of UGIH^21^. Therefore, in this multicentre case–control study, we conducted an exploratory analysis of the effect of eNOS intron 4 VNTR polymorphism on UGIH in users and non-users of NSAIDs (any NSAID, non-aspirin and aspirin NSAIDs).

The findings of this study reveal that genetic variants of e-NOS intron 4 VNTR polymorphism might not interact with aspirin and consequently, they might not modify the odds of UGIH in aspirin users. Our results also suggest a potentially decreased odds of UGIH in users of non-aspirin NSAIDs who carry the polymorphic genotype of e-NOS intron 4 VNTR. The interaction between non-aspirin NSAIDs and e-NOS intron 4 VNTR polymorphism was not statistically significant, hence, these secondary findings should be considered exploratory and interpreted with caution until replicated in a larger study. If these findings were confirmed, it would mean that the odds of UGIH in any NSAID users carrying e-NOS intron 4 VNTR polymorphism could be overestimated due to the inclusion of aspirin users. They would also allow better management of non-aspirin NSAID personalized medicine.

At the molecular level, the production of NO, a mediator of various physiological functions in the gastrointestinal tract^27^, seems to be affected by the genetic variation of 27-bp VNTR of e-NOS intron 4. The microRNA derived from this VNTR polymorphism suppresses e-NOS expression, and in specific the ancestral allele “4b” generates more microRNA and thus inhibits further the production of NO^24,50,51^. On the contrary, the intake of low-dose aspirin increases NO production by blood vessels and stimulates e-NOS enzymatic activity^52^. As e-NOS is the main isoform responsible for the regulation of vasodilation in the gastrointestinal system, it is expected that carrying the allelic variant 4a of e-NOS may reduce the risk of gastrointestinal bleeding. However, our exploratory analysis revealed that e-NOS intron 4 VNTR polymorphism is not associated with reduced odds of bleeding in aspirin users. This finding could be influenced by the limited number of individuals with e-NOS intron 4 VNTR polymorphism who were on aspirin treatment in our study. Due to the lack of full-case control studies (i.e., studies that encompass users and non-users of aspirin), we cannot compare our findings with those of other reports. However, it is crucial to highlight that our results differ from that of Piazuelo and colleagues who found an association between the genetic variant “4a” of e-NOS gene and lower odds of UGIH in aspirin users^32^. The study of Piazuelo involved aspirin users exclusively, and thus the effect of 4a e-NOS allele on aspirin-related UGIH could not be determined^53^. Accordingly, future larger studies are required to guarantee a comprehensive assessment of the effect of e-NOS intron 4 VNTR polymorphism on UGIH in users of aspirin and NSAIDs.

In general, the use of VNTR polymorphism in NSAID or aspirin personalized medicine has been very limited so far, although the high polymorphic characteristics of VNTR make them very informative in determining the loci for diseases^31^. Research studies have mainly focused on investigating the effect of SNPs in complex diseases or studying drug responses^54^. To date, only five studies have examined the association of VNTR polymorphism with aspirin and/or NSAIDs. These studies were carried out in different populations and targeted distinct health conditions, highlighting therefore the need for more research on the influence of VNTR polymorphism on NSAIDs- and aspirin-related UGIH (Table 4).

**Table 4:** Summary of studies characteristics about the interaction between VNTRs and aspirin or NSAIDs.

Study	Country	Patients’ characteristics	Health condition	Gene	Drug-gene interaction analysis
Kleinstein et al.^55^	United States	NSAIDs exposed and nonexposed participants	Colorectal carcinogenesis	ALOX5 and FLAP poly A promoter repeat [-169(PolyA)]	Yes
Piazuelo et al.^32^	Spain	Aspirin exposed participants	UGIH	eNOS	No
Poole et al.^56^	United States	NSAIDs/aspirin exposed and nonexposed participants	Colorectal polyps	PGIS and ALOX5	Yes
Jin et al.^57^	China	Aspirin exposed participants	Aspirin resistance	glycoprotein Ib α	No
Cervera el al.^58^	Spain	Aspirin exposed participants	Aspirin treatment failure against Ischemic stroke	glycoprotein Ib α	No

ALOX5, arachidonate 5-lipoxygenase; eNOS, endothelial nitric oxide synthase; FLAP, 5-lipoxygenase-activating protein; PGIS, prostacyclin synthase; UGIH, upper gastrointestinal haemorrhage.

Though our study is exploratory, it is characterized by various points of strength that might help designing future larger studies. Including both groups of participants “exposed and unexposed to NSAIDs” and stratifying by the type of NSAIDs (i.e., aspirin and non-aspirin) permitted providing a comprehensive assessment of these drugs. Adjusting the measures of effect for a large number of potentially confounding factors decreased the risk of confounding in these estimates^59^. Limiting the analysis to a specific ethnic group (i.e., using the native tongue of the participant or the original mother/father language as a proxy of ethnicity) allowed avoiding bias due to ethnic differences. The inclusion of only biologically unrelated participants prevented bias from the over-representation of ancestral alleles^60^. The use of catalogue of NSAIDs prompt cards during the interview aided the participants to recall and identify better the use of these drugs, and reviewing the medical records of the patients assisted in the reduction of the risk of recall bias.

Our findings need to be confirmed by larger studies and in different populations. Stratifying the study population by drug exposure and genotype resulted in a limited number of UGIH cases and controls in each stratum, especially in aspirin subgroup analysis, which was then reflected in the width of the estimated confidence intervals. The sample size limitation also precluded carrying out the initially planned dose–response analysis. Therefore, our findings correspond to any exposure to NSAIDs/aspirin. Reproducing the study in a larger population would allow for (1) increasing the number of cases and controls per subgroup and consequently improving the statistical power of the observed associations and (2) undertaking further analysis such as evaluating a dose–response relationship. Furthermore, our results cannot be generalized to non-white European populations due to ethnic differences. Replicating the study in different ethnic groups would permit testing the association between e-NOS intron 4 VNTR polymorphism, NSAIDs and UGIH in genetically different populations.

In conclusion, this exploratory study suggests that exposure to NSAIDs or aspirin does not modify the odds of UGIH according to patient’s e-NOS intron 4 genotype. It also suggests that e-NOS intron 4 VNTR polymorphism might reduce the odds of UGIH in non-aspirin NSAID users. More research is needed to understand the pharmacogenetics of NSAIDs, especially aspirin, which possess a large spectrum of protective and prophylactic properties, and more room should be given to VNTR polymorphism in studies oriented towards personalized medicine.

## Data Availability

The datasets generated and analyzed for this study can be found in the FigShare Repository [https://doi.org/10.6084/m9.figshare.13109663.v1].
